# Supplementary material for: Investigation on cement-improved phyllite based on the vertical vibration compaction method
Source: PLoS One. 2021 Mar 3;16(3):e0247599. doi: 10.1371/journal.pone.0247599 (PMC7928446; doi:10.1371/journal.pone.0247599)
Supplement: S1 Data — (DOCX) [file pone.0247599.s001.docx]

Data used in Fig 2.

Comparison of maximum dry density and optimal water content between heavy compaction and VVCM tests

| Phyllite type | Compaction method | Water content（%） | | | | | |
| --- | --- | --- | --- | --- | --- | --- | --- |
|  |  | 2 | 4 | 6 | 8 | 10 | 12 |
| Phyllite A | VVCM | 2.205 | 2.239 | 2.268 | 2.249 | 2.190 | - |
|  | Heavy compaction method | - | 2.162 | 2.205 | 2.208 | 2.164 | 2.103 |
| Phyllite B | VVCM | - | 2.177 | 2.208 | 2.230 | 2.154 | 2.135 |
|  | Heavy compaction method | - | 2.119 | 2.157 | 2.182 | 2.142 | 2.096 |
| Phyllite C | VVCM | - | 2.146 | 2.204 | 2.219 | 2.166 | 2.116 |
|  | Heavy compaction method | - | 2.039 | 2.103 | 2.151 | 2.132 | 2.102 |

Data used in Fig 3.

The relationship between *E*_c_ and compactness of the weathered phyllite

| Phyllite A | compactness（%） | 93.0 | 96.5 | 98.4 | 99.8 | 100.8 |
| --- | --- | --- | --- | --- | --- | --- |
|  | *E*_c_（MPa） | 42.5 | 53.6 | 65.1 | 72.5 | 77.9 |
| Phyllite B | compactness（%） | 93.5 | 96.2 | 98.8 | 99.6 | 100.5 |
|  | *E*_c_（MPa） | 33.4 | 45.7 | 50.7 | 53.5 | 57.3 |
| Phyllite C | compactness（%） | 92.8 | 95.9 | 98.1 | 99.8 | 100.7 |
|  | *E*_c_（MPa） | 20.9 | 26.1 | 29.6 | 34.5 | 38.3 |

Data used in Fig 4.

*CBR* of the weathered phyllite samples under different compaction methods

| Compaction method | Phyllite A | | Phyllite B | | Phyllite C | |
| --- | --- | --- | --- | --- | --- | --- |
|  | compactness（%） | *CBR*（%） | compactness（%） | *CBR*（%） | compactness（%） | *CBR*（%） |
| VVCM | 93.0 | 6.8 | 93.1 | 5.2 | 93.7 | 2 |
|  | 94.7 | 7.5 | 95.4 | 5.7 | 94.1 | 2.2 |
|  | 96.9 | 8.9 | 97.4 | 6.3 | 96.2 | 3 |
|  | 98.5 | 10.7 | 98.5 | 6.9 | 98.0 | 3.8 |
|  | 99.5 | 11.3 | 99.8 | 7.8 | 99.1 | 4.4 |
| Heavy compaction method | 92.9 | 6.3 | 92.4 | 4.2 | 93.1 | 1.7 |
|  | 94.8 | 6.9 | 94.3 | 4.4 | 94.7 | 2.1 |
|  | 96.4 | 7.5 | 95.8 | 4.7 | 95.7 | 2.4 |
|  | 98.1 | 8.4 | 97.8 | 4.9 | 98.4 | 3.2 |
|  | 99.4 | 10.1 | 99.1 | 5.3 | 99.3 | 3.4 |

Data used in Fig 5.

Relationship between CBR and compactness of phyllites

| Phyllite type | compactness（%） | CBR (%) under different overburden pressure (kPa) | | |
| --- | --- | --- | --- | --- |
|  |  | 10 | 20 | 30 |
| Phyllite A | 92.9 | 6.3 | 8.6 | 10 |
|  | 94.8 | 6.9 | 9.4 | 10.8 |
|  | 96.4 | 7.5 | 9.9 | 11.4 |
|  | 98.1 | 8.4 | 10.6 | 12.3 |
|  | 99.4 | 10.1 | 12.3 | 13.9 |
| Phyllite B | 92.4 | 4.2 | 5.4 | 6.1 |
|  | 94.3 | 4.4 | 5.6 | 6.3 |
|  | 95.8 | 4.7 | 5.8 | 6.5 |
|  | 97.8 | 4.9 | 6.1 | 6.8 |
|  | 99.1 | 5.3 | 6.7 | 7.4 |
| Phyllite C | 93.1 | 1.7 | 1.8 | 2 |
|  | 94.7 | 2.1 | 2.3 | 2.6 |
|  | 95.7 | 2.4 | 2.7 | 2.9 |
|  | 98.4 | 3.2 | 3.5 | 3.7 |
|  | 99.3 | 3.4 | 3.9 | 4.5 |

Data used in Fig 6.

Comparison of VVCM and heavy compaction method

| Phyllite type | Cement content（%） | VVCM | | Heavy compaction method | |
| --- | --- | --- | --- | --- | --- |
|  |  | Optimal water content（%） | Maximum dry density（g/cm^3^） | Optimal water content（%） | Maximum dry density（g/cm^3^） |
| Phyllite A | 2 | 5.8 | 2.271 | 6.1 | 2.250 |
|  | 3 | 6.1 | 2.277 | 6.2 | 2.254 |
|  | 4 | 6.2 | 2.284 | 6.4 | 2.257 |
|  | 5 | 6.5 | 2.288 | 6.6 | 2.262 |
| Phyllite B | 2 | 7.0 | 2.203 | 7.2 | 2.179 |
|  | 3 | 7.4 | 2.215 | 7.6 | 2.182 |
|  | 4 | 7.9 | 2.218 | 8.2 | 2.189 |
|  | 5 | 8.4 | 2.222 | 8.8 | 2.191 |
| Phyllite C | 2 | 8.2 | 2.171 | 8.3 | 2.143 |
|  | 3 | 8.4 | 2.176 | 8.6 | 2.146 |
|  | 4 | 8.8 | 2.180 | 9.0 | 2.150 |
|  | 5 | 9.2 | 2.183 | 9.3 | 2.153 |

Data used in Fig 7. Fig 8. Fig 9.

Fig 7. Influence of cement content on *R*_c_

Fig 8. The relationship between 7d *R*_c_ and compactness

Fig 9. Relationship between 7d *R*_c_ and compaction method

| Phyllite type | Compaction method | compactness  （%） | 7d *R*_c_ (MPa) of improved phyllite under different cement content (%) | | | |
| --- | --- | --- | --- | --- | --- | --- |
|  |  |  | 2 | 3 | 4 | 5 |
| Phyllite A | VVCM | 95 | 0.59 | 0.92 | 1.06 | 1.38 |
|  |  | 97 | 0.76 | 1.03 | 1.35 | 1.69 |
|  |  | 99 | 0.88 | 1.21 | 1.49 | 1.95 |
|  |  | 101 | 0.96 | 1.30 | 1.62 | 2.04 |
|  |  | 103 | 1.20 | 1.44 | 1.86 | 2.26 |
|  | Static pressure method | 95 | 0.48 | 0.85 | 1.01 | 1.18 |
|  |  | 97 | 0.62 | 1.06 | 1.27 | 1.46 |
|  |  | 99 | 0.86 | 1.07 | 1.31 | 1.69 |
|  |  | 101 | 0.96 | 1.21 | 1.45 | 1.87 |
|  |  | 103 | 1.08 | 1.35 | 1.66 | 2.01 |
| Phyllite B | VVCM | 95 | 0.47 | 0.60 | 0.72 | 0.89 |
|  |  | 97 | 0.66 | 0.74 | 0.90 | 1.15 |
|  |  | 99 | 0.75 | 0.86 | 1.16 | 1.37 |
|  |  | 101 | 0.82 | 1.15 | 1.38 | 1.61 |
|  |  | 103 | 1.16 | 1.37 | 1.59 | 1.83 |
|  | Static pressure method | 95 | 0.41 | 0.52 | 0.61 | 0.78 |
|  |  | 97 | 0.58 | 0.69 | 0.75 | 0.81 |
|  |  | 99 | 0.61 | 0.78 | 1.07 | 1.18 |
|  |  | 101 | 0.82 | 1.08 | 1.25 | 1.36 |
|  |  | 103 | 1.02 | 1.19 | 1.40 | 1.65 |
| Phyllite C | VVCM | 95 | 0.44 | 0.56 | 0.67 | 0.81 |
|  |  | 97 | 0.60 | 0.74 | 0.83 | 0.93 |
|  |  | 99 | 0.65 | 0.83 | 0.97 | 1.20 |
|  |  | 101 | 0.78 | 0.91 | 1.23 | 1.31 |
|  |  | 103 | 0.95 | 1.10 | 1.33 | 1.56 |
|  | Static pressure method | 95 | 0.39 | 0.46 | 0.54 | 0.62 |
|  |  | 97 | 0.54 | 0.67 | 0.77 | 0.79 |
|  |  | 99 | 0.59 | 0.75 | 0.92 | 0.95 |
|  |  | 101 | 0.61 | 0.78 | 1.07 | 1.18 |
|  |  | 103 | 0.88 | 0.98 | 1.21 | 1.35 |

Data used in Fig 10. Fig 11. Fig 12.

Fig 10. The influence of cement content on *E*_c_

Fig 11. Relationship between 7d *E*_c_ and compactness

Fig 12. The relationship between 7d *E*_c_ and compaction method

| Phyllite type | Compaction method | compactness  （%） | 7d *E*_c_ (MPa) of improved phyllite under different cement content (%) | | | |
| --- | --- | --- | --- | --- | --- | --- |
|  |  |  | 2 | 3 | 4 | 5 |
| Phyllite A | VVCM | 95 | 104 | 122 | 174 | 209 |
|  |  | 97 | 120 | 169 | 217 | 254 |
|  |  | 99 | 147 | 201 | 243 | 272 |
|  |  | 101 | 162 | 223 | 262 | 325 |
|  |  | 103 | 171 | 254 | 312 | 359 |
|  | Static pressure method | 95 | 95 | 112 | 153 | 178 |
|  |  | 97 | 113 | 158 | 189 | 221 |
|  |  | 99 | 129 | 183 | 211 | 236 |
|  |  | 101 | 148 | 202 | 235 | 287 |
|  |  | 103 | 159 | 235 | 268 | 303 |
| Phyllite B | VVCM | 95 | 74 | 89 | 109 | 122 |
|  |  | 97 | 95 | 136 | 154 | 171 |
|  |  | 99 | 103 | 139 | 170 | 204 |
|  |  | 101 | 119 | 153 | 203 | 242 |
|  |  | 103 | 131 | 189 | 222 | 276 |
|  | Static pressure method | 95 | 68 | 81 | 99 | 115 |
|  |  | 97 | 87 | 121 | 137 | 146 |
|  |  | 99 | 94 | 132 | 148 | 165 |
|  |  | 101 | 109 | 147 | 186 | 204 |
|  |  | 103 | 120 | 169 | 203 | 231 |
| Phyllite C | VVCM | 95 | 61 | 76 | 90 | 101 |
|  |  | 97 | 64 | 80 | 96 | 112 |
|  |  | 99 | 69 | 85 | 108 | 125 |
|  |  | 101 | 78 | 96 | 118 | 146 |
|  |  | 103 | 97 | 110 | 132 | 157 |
|  | Static pressure method | 95 | 56 | 73 | 80 | 89 |
|  |  | 97 | 58 | 77 | 88 | 93 |
|  |  | 99 | 63 | 80 | 90 | 101 |
|  |  | 101 | 71 | 90 | 102 | 126 |
|  |  | 103 | 89 | 105 | 118 | 135 |
