# Supplementary material for: Investigation on cement-improved phyllite based on the vertical vibration compaction method
Source: PLoS One. 2021 Mar 3;16(3):e0247599. doi: 10.1371/journal.pone.0247599 (PMC7928446; doi:10.1371/journal.pone.0247599)
Supplement: Attachment — Submitted filename: Response to Reviewers.docx [file pone.0247599.s003.docx]

Dear editor,

First of all, thank you and all reviewers for your recognition of my article. I have numbered the sections and subsections uniformly in accordance with the requirements of reviewer 5.

Dear reviewer 5,

Thank you again for reviewing and evaluating my manuscript, and the following is my reply and description.

Question: Apply unified numbering system for the sections and subsections.

**Response:** Thanks for the reviewer’s comments. Sorry for this problem. In the first submission of the manuscript, I gave a unified number to the sections, but I saw that the article templates on the website did not use a unified number, so I deleted it in the second submission. This point you raised is very valuable. Without a uniform section number, the article will appear unorganized. I have added it to the manuscript.
